# Assessing the role of physical activity in shaping students’ academic motivation: the mediating role of mental health

**DOI:** 10.1186/s12889-025-25541-8

**Published:** 2025-11-28

**Authors:** Dongmin Ma, Huma Akram, Shengji Li

**Affiliations:** 1 School of Foreign Studies, North China University of Water Resources and Electric Power, Zhengzhou, Henan China; 2 School of International Education, North China University of Water Resources and Electric Power, Zhengzhou, Henan China

**Keywords:** Physical activity, Mental health, Self-determination theory, Higher education, Intrinsic motivation, Extrinsic motivation

## Abstract

**Background:**

Student motivation and their mental wellness are key determinants of academic success in higher education. Among diverse strategies to support these outcomes, engagement in physical activity emerges as a particularly effective approach for promoting students’ psychological well-being and sustaining their motivation. To further examine a complex interplay among these variables, we investigated the influence of physical activity (PA) on students’ academic motivation (AM) through their mental health (MH) in light of self-determination theory (SDT).

**Methods:**

Implementing a quasi-experimental research design, we recruited 450 students and engaged them in a daily running practice for four months to examine its impact on their MH and AM. We assessed AM using an adapted version of the academic motivation scale (AMS), MH using the mental health and well-being scale (MWBS), and PA using the adapted version of the physical activity questionnaire for adolescents (PAQ-A) at three time points: prior to, midway through, and following the intervention. We analyzed the data both descriptively (via mean and standard deviation) and inferentially, employing two-way repeated measures ANOVA and structural equation modeling as our statistical approaches.

**Results:**

The intervention substantially improved students’ MH and AM, with extrinsic motivation (EM) consistently surpassing intrinsic motivation (IM) across all time intervals. The study also demonstrates a substantial influence of PA through MH on AM over time, with a greater mediating effect on EM (*ꞵ*= 0.17, *p* < 0.01) than IM (ꞵ= 0.12, *p* = 0.01). The findings suggest that students are more influenced by external motivators, such as grades and their career projections, than by the intrinsic enjoyment of learning.

**Conclusions:**

The consistent dominance of EM underscores the need for educational strategies that balance external incentives with opportunities to foster IM, emphasizing autonomy, competence, and personal growth. Additionally, the positive impact of running practice through MH on both types of motivation suggests that incorporating psychological support into regular PA programs can enhance student academic engagement and psychological wellbeing. The findings further suggest ways to meet the intrinsic needs of students to cultivate their internal desire to learn.

## Introduction

Considering the demand of the present age, students’ academic progress in higher education holds a significant position in educational research [1, 2]. In acknowledgment of this, the worldwide movement has given student engagement in higher learning a priority [3, 4], paying special attention to academic motivation [5, 6]. Academic motivation is the overarching term for the factors that influence students’ behaviours in the classroom, including their level of effort, their ability to self-regulate, and their resilience in the face of adversity [7]. It encompasses both intrinsic and extrinsic elements that drive students to engage with their studies, overcome challenges, and achieve their educational objectives [8]. Its importance in empowering students with self-monitoring and metacognition skills is considered crucial [9], which helps improve their cognitive abilities [10], academic involvement [11], capabilities of overcoming academic challenges [8], and desirable academic outcomes [12]. The motivational profiles of students can be attributed to several factors, such as their cultural background [13], cognitive abilities [14], educational experiences [15], social support [16], and mental health [2]. Among these, mental well-being has been considered vital in determining students’ ability to engage with academic tasks [17], sustain effort [18], and persevere through challenges [19]. When students experience positive mental health, they are more likely to exhibit self-confidence [20], emotional resilience [21], a strong sense of purpose [22], and sustain long-term motivation [23]. Conversely, students struggling with psychological problems such as anxiety and stress can severely hinder their interest in learning [24] by impairing their concentration and reducing energy levels [25]. Therefore, augmenting a favorable mental health environment has been deemed important for enhancing student motivation.

Given their importance, previous literature has used a broad array of interventions to strengthen students’ motivation and mental health, which are largely related to teaching practices [26], psychological counselling [27], social support programs [28], and extracurricular activities [29]. In the meantime, physical activity has been well-known for its major role in individual overall well-being [30], physical fitness [31], and psychological health [32]. Practicing it on a daily basis can decrease the secretion of stress-producing chemicals like adrenaline and cortisol, enhancing positive mood [33]. Among the several ways of exercise, running captures attention due to its ease, accessibility, and cost-effectiveness in educational settings. Engaging in it at a moderate intensity makes it suitable for gaining both physical and mental health benefits for a broad range of individuals [34]. Numerous studies have demonstrated that running’s beneficial properties increase mental health indicators, such as reducing negative emotional states [35] and anxiety [33] via releasing endorphins physiologically [30]. Empirical studies also link running with increased cognitive functioning [36] and self-efficacy [37], which supports students’ motivation to learn [38]. Yet, with all these interventions and studies, there is a gap about how physical activity (PA) relates to the students’ academic motivation (AM) in conjunction with mental health (MH). We thus intend to inquire into the benefits of running practice on the MH and motivational dynamics of students by following these objectives:

1. To look into the role of running practice in enhancing students’ mental health and academic motivation.
2. To analyze the mediating role of mental health between running practice and students’ academic motivation.

## Literature review

### Academic motivation

Academic motivation (AM) pertains to the internal drive, enthusiasm, and commitment of a person to his or her academic pursuits [6, 39]. Understanding its components and antecedents is critical for establishing efficient educational policies and initiatives to improve students’ academic performance. Analyzing its importance in educational attainment, Ryan and Deci [5] identify that AM is critical for sustaining students’ level of engagement and perseverance during learning activities. Likewise, surveying 365 students from five wide-ranging public universities, Karimi and Sotoodeh [40] identified that AM is positively related to students’ active participation. In another study, de la Fuente et al. [14] note that motivation fosters students’ cognitive abilities to solve academic problems efficiently as well as their abilities to regulate affective situations. In addition, previous studies indicate several factors that shape their motivation to engage in learning practices. For instance, surveying 437 students from diverse universities in Pakistan, Akram and Li [41] found that positive teacher-student associations had a substantial effect on students’ AM, with a greater effect on intrinsic motivation than extrinsic motivation. In addition, Bozgün and Akın-Kösterelioğlu [16] identified subjective well-being as a central indicator in sustaining students’ motivation in reading and writing in their quantitative analysis. Regarding barriers, several studies have reported psychological and physical factors as important hurdles that undermine students’ motivation to learn, such as depressive symptoms [15], emotional stress [42], physical [31], and mental wellness [2]. Taking together the above-mentioned barriers, the importance of physical exercise in coping with such issues is also well documented.

For instance, Kotaman and Evran [43] underline the benefits of being physically active for students, where they find themselves capable of sustaining their motivation to learn in a more positive manner. Adopting a quantitative approach, Aung et al.’s [44] study revealed a positive association of exercise behavior with students’ intrinsic academic motivation, suggesting that students to participate in physical activities. Linking PA with psychological and physical health benefits, Young-Jones et al. [31] conducted an experimental study, where they divided 209 college students into four different groups. The results demonstrated that students’ motivation and energy levels were positively impacted by moderate exercise and yoga interventions when contrasted with the control group. Considering the aforementioned studies, it is evident that exercising is considered a pleasurable activity. Despite these efforts, research on the association of PA in the conjugation with MH and AM in students remains in its early stages.

### Mental health

Mental health (MH) in the context of students pertains to their emotional resilience, enabling them to tackle everyday challenges, enjoy life fully, and maintain productivity throughout their educational journey [45]. It encompasses the belief in one’s own and others’ worth, which help in maintaining healthy relationships, coping with academic pressures, and fostering overall well-being. In exploring the importance of MH for achieving educational goals, Kim and Karr [22] highlight its critical role in enhancing academic performance. Their survey of 660 undergraduate students revealed a significant correlation between students’ growth mindset and their perceived academic self-efficacy. Similarly, Broglia and Barkham [17] underline the significance of mental fitness for students in meeting academic requirements and provide evidence-based recommendations for improving mental well-being. Regarding educational sustainability, numerous studies have examined the mental wellness of students in relation to enhancing academic performance [21], addressing educational issues [23], adapting to new educational settings [46], strengthening social connections [47], fostering cognitive abilities [48], and developing interprofessional collaborative skills [20]. Collectively, these perspectives underscore that MH is a crucial predictor of increased educational engagement and academic success.

Recognizing its importance, the National College Health Assessment (NCHA) [49] emphasizes the need to address students’ MH issues to support their educational objectives. Their findings indicate that approximately 50% of students experience depression and anxiety during their academic pursuits, largely due to the pressures associated with academic responsibilities. Brown [50] further illustrates that these challenges are most prevalent among students aged 18 to 25, a group that constitutes 80% of full-time undergraduates. To address these concerns, previous literature recommends a wide range of interventions to improve the MH of students, including mindfulness practices [42], counseling services [45], and physical exercise [32]. The psychological advantages of physical activities are widely acknowledged, contributing positively to individual psychological well-being [51], and cognitive function [52]. Regular engagement in these activities can improve mood [30], reduce symptoms of depression [53], promote relaxation [54], and develop emotional stability [35]. Physiologically, it helps in producing mood-related neurotransmitter hormones that boost positive feelings [55]. Furthermore, consistent PA can improve neurological function and strengthen cognitive skills, ultimately leading to improved educational outcomes [54]. Given that, we can propose the following hypothesis:

1. Running practice positively affects students’ mental health.

### Theoretical basis

**Fig. 1:**
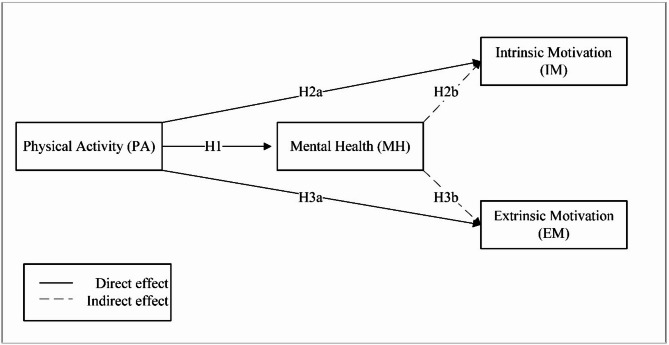
Conceptual model

Expanding on the study’s objectives, this study uses the conceptual framework of self-determination (SDT) theory [39, 56] to elucidate the motivational dynamics among learners (see Fig. 1). SDT provides a nuanced understanding of both intrinsic and extrinsic motivation, focusing on the driving forces behind individuals’ actions.

#### Intrinsic motivation

Intrinsic motivation (IM) can be inferred to an individual’s inherent interest to participate in an activity for its own sake, without the expectation of external rewards [57]. More specifically, this theory suggests that when an individual’s three basic psychological demands, i.e., competence, connection, and autonomy, are fulfilled, they become self-determined or internally motivated. Autonomy is characterized as the freedom to pursue one’s own interests; competence relates to an individual’s belief in their ability to perform tasks, and relatedness encompasses feelings of belonging and social connection [39, 56]. An academic environment that helps satisfy these psychological needs develops students’ self-regulatory abilities, leading to increased IM to learn [7, 7]. Conversely, an environment that doesn’t support the satisfaction of these needs may hinder their interests and IM [7]. In relation to physical activity, it serves as an intrinsic motivator that can enhance students’ IM by aligning running activity physical education with the three basic psychological needs [58]. Ahmadi et al. [59] further indicate through a meta-analysis that PA programs that empower students to control their physical activities and encourage social interaction, facilitate the acquisition of competency within those programs. Expanding on SDT, Tapia-Serrano et al. [60] specify the positive effects of IM in primary education, observing through a longitudinal study that SDT-integrated PA approaches bolster students’ IM toward PA behaviors.

Regarding the role of PA, de Bruijn et al. [61] suggest IM and academic achievement as crucial predictors of success in physical education. Their study, which involved 891 students participating in PA interventions over 14 weeks, demonstrated that the level of student engagement in physical education significantly influences their motivation to learn. Additionally, Hizomi Arani et al. [62] found that consistent involvement in PA programs improves individual’s fulfillment and self-efficacy, both of which are the core determinants of IM. The positive effects of PA on MH are also recognized widely. Smith and Fernhall [32] illustrate that PA practices can alleviate symptoms of depression, thereby enhancing students’ cognitive abilities and laying the groundwork for self-directed learning. Likewise, McBride and Greeson [48] assert that good MH is vital for maintaining students’ interest in their educational pursuits. In light of this, these hypotheses can be projected in our study’s context:

- Running practice positively affects students’ intrinsic motivation (IM).
- Mental health mediates the relationship between running practice and students’ IM.

#### Extrinsic motivation

The second type, extrinsic motivation (EM), alludes to engaging individuals in activities where their behavior is influenced by external factors [39, 56]. These factors can be tangible, such as money or grades, or intangible, such as respect or appreciation. More specifically, individuals who are influenced by external factors are more likely to engage in activities even if they do not enjoy them [63]. For instance, a student may compel themselves to study hard for exams, despite finding the process undesirable. Expanding on this concept, Zhou and Zhang [64] specify that EM is a component of operant conditioning, where an individual or object undergoes conditioning in a specific way through positive or negative outcome. Linking EM to IM, Ryan and Deci [5] found that students who recognized the value of academic tasks, exhibited higher levels of engagement and persistence, even when their initial motivation was extrinsic. Investigating this further, Razali et al. [65] looked at the role of the Quizizz application on students’ motivation, and revealed that when teachers align learning practices with competitive and reward-based games, students are more likely to adopt these tasks, leading to more self-determined forms of EM. Regarding PA, it can be considered as an external motivator that can boost students’ EM by linking these practices to tangible rewards and encouragement from others [66]. School-based PA programs exemplify organized activities where participation, effort, and achievement can earn students’ grades, certificates, or public recognition. Building on this conceptualization, a meta-analysis carried out by Kelso et al. [52] identified the importance of external rewards in increasing students’ motivation to participate in both physical and academic activities. Similarly, a study conducted by Kamal and Zulkifli [67] found that when students receive rewards for participating in PA programs, they are more likely to exhibit similar levels of effort and persistence in their academic pursuits. This suggests that the positive encouragement they gain through PA programs may translate into their educational activities, potentially leading to improved learning outcomes. Given this, we can anticipate the following hypotheses:

- Running practice positively affects students’ extrinsic motivation (EM) over time.
- Mental health mediates the relationship between running practice and students’ EM.

## Methodology

We introduced a moderate intensity running exercise at a Chinese university using a quasi-experimental research method to examine its impact on students’ MH and AM. The design of this intervention was aligned with SDT [39, 56] to support students’ core psychological needs, i.e., autonomy, competence, and relatedness. Firstly, we allowed students to choose their own running pace and schedule within a given timeframe to support their autonomy. We then instructed them to track their own progress on a weekly basis to gradually increase their efficiency. Furthermore, they were encouraged to run in small peer groups to develop feelings of connection among them. The recruiting period for this study began on September 1, 2023, and lasted until December 31, 2023. To assess the changes over time, the pre-test was administered at the beginning of the intervention, specifically on September 1. The mid-test was conducted on October 30, and the post-test was held on December 31, 2023. Given the structured nature of the study, we selected 450 undergraduate students from various academic disciplines and educational levels using a convenience sampling procedure. This approach is particularly suitable for intervention-based studies because it facilitates the easy recruitment of participants from the same location [68]. It also streamlines coordination, enabling the researcher to collect data quickly and efficiently. At the same time, it restricts the generalizability of findings, suggesting that results should be interpreted cautiously in relation to other cultural contexts. Students with medical problems that would hinder the proper practice of PA were excluded to effectively examine the intervention’s effect. Although we did not calculate the sample size beforehand, a post hoc power analysis was conducted using G*Power 3.1.9.7 to evaluate the adequacy of the sample size. This analysis demonstrated a significant result with a power of 0.96 [69], indicating sufficient power to detect the observed effects. To ensure the equivalence of participants’ traits, baseline comparisons were conducted using independent t-tests and chi-square tests before launching the intervention.

After receiving approval from the institutional review board at North China University of Water Resources and Electric Power, we had students participate in running practice on the university’s playground for thirty minutes each day over a period of four months. The field offers a standard 400-meter track with sufficient space to accommodate participants while ensuring their safety and comfort. Taking into account students’ preferences, a structured timeframe from 8 a.m. to 5 p.m. was established to allow them to select their preferred running time. Additionally, they were encouraged to run at a pace that enabled them to maintain communication, harmonizing with the WHO’s [34] recommended moderate-intensity range. Their attendance was tracked via a digital form with an embedded timestamp on WeChat, accessible via a QR code placed at the designated track to guarantee the study’s validity. Each participant was supposed to check in before running and then check out after completion. For verification, rotating shifts of research assistants were present throughout the day to supervise and record attendance. All students took part in the study willingly and provided oral informed consent before participating in the study. They were also made aware of their rights to stop participating at any point during the study. The online survey consisted of an introductory message that explained the objective of the study and ensured each participant’s confidentiality. Furthermore, each method of the project adhered to the pertinent regulations and Helsinki’s human experimentation rules.

### Measure

We employed different measures at three time points, i.e., prior, mid, and after the intervention, using a closed-ended questionnaire to assess the key constructs of the study. The questionnaire was provided in both Chinese and English to ensure that all students could comprehend it effectively. To further enhance the study’s contextual relevance, professional linguists examined the questionnaire to confirm participants’ understanding and response precision, thereby guaranteeing content quality and consistency [70]. Participants were then given access to the survey through the WeChat mobile application at each evaluation point.

#### Academic motivation (AMS)

Students’ academic motivation was assessed using an adapted version of Guay et al.’s [71] academic motivation scale (AMS) to align with the study design. The original scale consisted of 28 items that were organized into three main constructs. The first construct, intrinsic motivation (IM), included three subscales: IM to know (mic), IM to stimulation (mis), and IM to accomplishment (mia). The second construct, extrinsic motivation (EM), also had three subscales: identified regulation (iden), introjected regulation (Intro), and external regulation (Reg), while third construct was amotivation (Amo). Following the scale evaluation by Guay et al. [71], we removed six items (i.e., mic4, mis4, mia4, iden4, Intro4, and Reg4) due to low factor loadings (ranging from.29 to.42), along with the four items from the amotivation subscale (amo1–amo4), as they were out of the scope of our study. The final adapted version comprised of 18 items with satisfactory factor loadings ranging from (.78 to.89), across two constructs: intrinsic motivation (IM) and extrinsic motivation (EM), with each representing by nine items. We merged the subscales of both constructs intrinsic and extrinsic motivation and relabeled the retained items for clarity as IM1–IM9 and EM1–EM9, respectively (see Table 3). In addition, we adapted the scale to a 5-point response format (ranging from “strongly disagree” to “strongly agree”) to align with the evaluation of the other measures, allowing researchers to compare interpretations across constructs. For interpretive purpose, we followed Alkharusi’s [72] prescribed guidelines, classifying scores from 1.0 to 2.5 as low motivation, scores from 2.6 to 3.4 as moderate, and scores from 3.5 to 5.0 as high motivation (applicable to both IM and EM). This scale’s authenticity has been substantiated by many studies across diverse contexts, such as Litalien et al. [11] in Canada and Howard et al. [73] in different countries. Items of both scales were slightly modified in relation to students’ experiences during or after running sessions, for instance, “Acquiring new knowledge brings me joy and contentment.”

#### Mental health (MWBS)

Students’ mental health was assessed through Stochl et al.’s [74] 14-item, mental health and well-being scale (MWBS) scale, which supports 5-point response options (ranging from “strongly disagree” to “strongly agree”). Following Alkharusi’s [72] guidelines, scores ranging from 1.0 to 2.5 were classified as low mental well-being, scores from 2.6 to 3.4 as moderate, and scores from 3.5 to 5.0 as high mental well-being. This scale’s authenticity has been substantiated by many studies in different settings, such as Li and Huang [75] in China. Items of the measure was slightly amended in relation to students’ experiences during or after running sessions, for instance, “I feel more optimistic about the future after starting a running practice.”

#### Physical activity (PAQ-A)

Physical activity engagement was assessed using Kowalski et al.’s [76], physical activity questionnaire for adolescents (PAQ-A), which utilized a 5-point response format ranging from “never” to “every day.” The original scale includes nine items assessing a wide range of physical activities. To investigate the actual impact of running practice on the study variables, we chose and modified three items that measure the intensity, frequency, and consistency of running practice, allowing us to utilize a 5-point response scale. The items included: (1) How often do you engage in running practice (e.g., jogging, steady running) in a typical week? (2) How many days you stayed engaged in running practice in the last 7 days? (3) How many evenings you stayed engaged in running practice in the last 7 days? Leveraging Alkharusi’s [72] criteria, scores from 1.0 to 2.5 were categorized as low physical activity engagement, scores from 2.6 to 3.4 as moderate engagement, and scores from 3.5 to 5.0 as high physical activity engagement. This scale has been validated in various contexts, including studies by Chacón-Cuberos et al. [30] in a Spanish context and Qin et al. [77] in a Chinese context. Its items were slightly modified to align with the running practice intervention of our study.

### Data analysis

All statistical analyses were conducted using IBM SPSS Statistics 28.0 and Smart PLS to examine the study’s objectives. We first used descriptive statistics (mean, standard deviation, frequencies) to compute participants’ demographics and scores of the study variables. Inferentially, we used two-way repeated measures ANOVA to evaluate the intervention’s effect over time. We then used structural equation modeling to examine the mediating role of MH in the relationship between PA and AM. We treated all constructs as latent variables and modeled PA as the predictor, MH as the mediator, and (intrinsic and extrinsic) motivation as the outcome. A significance threshold of *p* < 0.05 was used for all inferential analyses. Effect sizes were reported using partial eta squared (*η*²) for ANOVA results and standardized beta coefficients (β) for SEM paths to assess the magnitude of effects.

## Results

Before initiating the remedial program, we used a survey to confirm that the assigned respondents didn’t have experience with daily running to evaluate the effectiveness of the intervention. Out of 450 students, there were 298 males and 152 females among the participants, who ranged in age from 18 to 23. Regarding academic disciplines, 279 students were from the natural sciences and 171 from the social sciences. Concerning educational levels, 161 students belonged to the first-year, 123 to the second, while 166 to the third-year cohorts. We then compared these demographic traits and study variables statistically (see Table 1) to ensure uniformity. Continuous variables, i.e., age, PA, MH, IM, and EM, were evaluated via an independent sample t-test, while categorical variables, i.e., gender, academic discipline, and educational level, were evaluated via a chi-square test. The evaluation found insignificant differences across gender (*χ*² = 0.41, *p* = 0.09), age (*t* = 0.16, *p* = 0.14), academic discipline (*t* = 0.38, *p* = 0.11), educational level (*t* = 0.36, *p* = 0.15), and study’s variables, i.e., MH (*t* = 0.92, *p* = 0.14), IM (*t* = 1.14, *p* = 0.15), and EM (*t* = 0.93, *p* = 0.23). These results prove that all students were demographically equivalent at the start of the intervention.

**Table 1:** Comparison across demographic factors

Variables	Sub-categories	*N*	%/Mean (SD)	(t/χ2)	*p*-value
Gender	Male	298	66%	0.41	0.09
	Female	152	34%		
Age	18–20 years	250	20.4 (1.8)	0.16	0.14
	21–23 years	200			
Academic discipline	Natural Sciences	279	62%	0.38	0.11
	Social Sciences	171	38%		
Educational level	First-year	161	36%	0.36	0.15
	Second-year	123	27%		
	Third-year	166	37%		
MH	-	-	3.12 (0.88)	0.92	0.14
IM	-	-	2.55 (0.74)	1.14	0.15
EM	-	-	2.81 (0.77)	0.93	0.23

*MH* Mental Health, *IM* Intrinsic Motivation, *EM* Extrinsic Motivation, *SD* Standard Deviation, *p* probability value

Before analyzing data, confirmatory factor analysis (CFA) was used to assess the model’s suitability in the context of our study (see Table 2). According to the goodness-of-fit ratings, the model fit the data in a satisfactory way [78], confirming the robustness and dependability of the study instrument.

**Table 2:** Model fit matrix

Indicators	Threshold values	Model values
χ2/df	< 3	2.62
RMSR	< 0.08	0.75
RMSEA	< 0.08	0.072
CFI	> 0.90	0.94
GFI	> 0.90	0.94
AGFI	> 0.8	0.86
TLI	> 0.90	0.93

In addition, the reliability and validity of each dimension were assessed to ensure that the outcomes aligned with the study model (refer to Table 3). Cronbach’s alpha was used to assess the reliability of each parameter, and results above 0.80 demonstrated sufficient convergence to move forward with data analysis [79]. The validity indicators also yielded robust values, i.e., composite reliability CR >0.70, average variance extracted AVE >0.50, and discriminant validity DV >0.85 [80]. The construct validity was further elaborated by the factor loadings, which produced discriminant validity for all constructs [78] and high loadings for all items [81].

**Table 3:** Reliability and validity indices

Variables	Items	Factor Loadings	CR	Alpha Value	AVE	DV
Intrinsic Motivation (IM)	IM1	0.79	0.87	0.83	0.74	0.86
	IM2	0.78				
	IM3	0.82				
	IM4	0.81				
	IM5	0.80				
	IM6	0.81				
	IM7	0.83				
	IM8	0.82				
	IM9	0.82				
Extrinsic Motivation (EM)	EM1	0.80	0.88	0.82	0.73	0.85
	EM2	0.81				
	EM3	0.82				
	EM4	0.81				
	EM5	0.82				
	EM6	0.80				
	EM7	0.79				
	EM8	0.79				
	EM9	0.79				
Mental health (MH)	MH1	0.78	0.87	0.81	0.74	0.86
	MH2	0.79				
	MH3	0.80				
	MH4	0.80				
	MH5	0.81				
	MH6	0.80				
	MH7	0.79				
	MH8	0.79				
	MH9	0.89				
	MH9	0.80				
	MH10	0.81				
	MH11	0.81				
	MH12	0.80				
	MH13	0.80				
	MH14	0.82				
Physical Activity (PA)	PA1	0.80	0.86	0.81	0.77	0.87
	PA2	0.80				
	PA3	0.82				

*CR* Composite Reliability, *AVE* Average Variance Extracted, *DV *Discriminant Validity

**Fig. 2:**
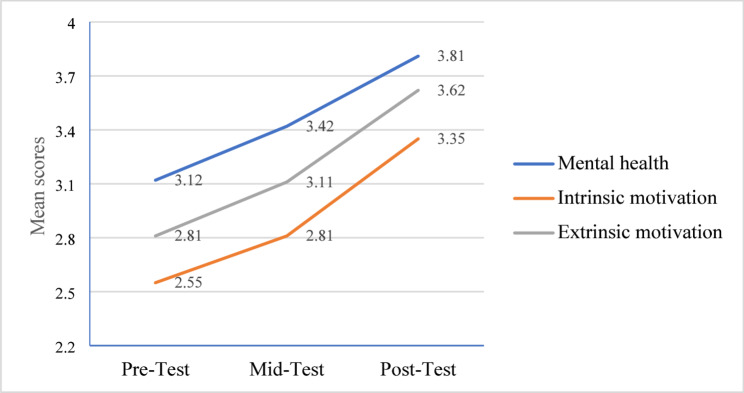
Mean values over time

Analyzing data, students’ academic motivation and mental health were evaluated at three time intervals descriptively, while the significance of the running intervention was assessed via two-way repeated measures ANOVA. The mediating role of mental health between running exercise and students’ academic motivation was further evaluated employing a structural equation model (SEM) approach via a bootstrapping procedure. Analyzing descriptively, we found significant variations in students’ academic motivation and mental health progressively (see Table 4). Regarding motivation, students had relatively low initial IM and EM levels, while mid-test results showed a slight upsurge in both constructs as the intervention went on (see Fig. 2). This upsurge in both IM and EM was maintained throughout the post-test stage, with EM exhibiting the major rise (M = 3.62) compared to IM (M = 3.11). We also observed a notable improvement in students’ MH over time, with a mean score of 3.81 at the post-test stage.

**Table 4:** Descriptive statistics

Time interval	MH		IM		EM	
	M	SD	M	SD	M	SD
Pre-Test	3.12	0.88	2.55	0.74	2.81	0.77
Mid-Test	3.42	0.89	2.81	0.75	3.11	0.80
Post-Test	3.81	0.82	3.35	0.89	3.62	0.86

*MH* Mental Health, *IM * Intrinsic Motivation, *EM* Extrinsic Motivation, *SD* Standard Deviation, *M * Mean

To observe the intervention time’s effect on the academic (intrinsic and extrinsic) motivation and mental health of students, a two-way repeated measures ANOVA was conducted (see Table 5). Main effects revealed significant improvements across all constructs from pre- to post-intervention, F(2, 898) = 41.23, *p* = < 0.001, *η²* = 0.32. With a 95% confidence interval of 1.50 for the aggregate mean difference, this result supports hypotheses 1, 2a, and 3a, confirming that the running-based intervention positively influenced students’ overall psychological and motivational outcomes. In terms of strength, the main effect of time was strongest for mental health, F(2, 898) = 31.11, *p* < 0.001, *η²* = 0.32, with a mean difference of 1.40 (95% CI), while IM demonstrated comparatively smaller, improvement, *F*(2, 898) = 26.12, *p* = 0.01, *η²* = 0.23 than other constructs.

**Table 5:** Repeated measures ANOVA matrix

Constructs/Effect	SS	df	MS	F	*p*	*η²*
Time	69.21	2	35.11	41.23	0.00**	0.32
IM	20.24	2	15.12	26.12	0.01*	0.23
Time × IM	18.02	4	4.37	13.21	0.00**	0.11
EM	21.31	2	12.19	29.16	0.00**	0.29
Time × EM	19.04	4	4.13	15.14	0.00**	0.14
MH	22.23	2	9.13	31.11	0.00**	0.32
Time × MH	21.22	4	4.15	18.22	0.00**	0.15
Error (Residual)	93.23	65	0.99	-	-	-

*SS* Sum of Squares, *df* Degrees of Freedom, *MS* Mean Square, *F* F-statistic, *p* Significance level, *η²* Partial Eta Squared (Effect Size), *MH* Mental Health, *IM* Intrinsic Motivation, *EM* Extrinsic Motivation

Significant at: *p < 0.05, **p < 0.01

The results regarding interaction effects indicated significant improvements across all constructs, indicating that the degree of change varied among these variables over time. Specifically, the interaction effect was strongest for mental health, with F(4, 898) = 18.22, *p* < 0.001, *η²* = 0.15, whereas the improvement in IM was comparatively smaller, i.e., *F*(2, 898) = 13.21, *p* = < 0.001, *η²* = 0.11, than in the other constructs. Moreover, using the Bonferroni correction, post-hoc comparisons between groups indicated significant differences across all constructs in every phase, indicating a notable enhancement from the beginning to the post-intervention (all p-values < 0.05).

**Fig. 3:**
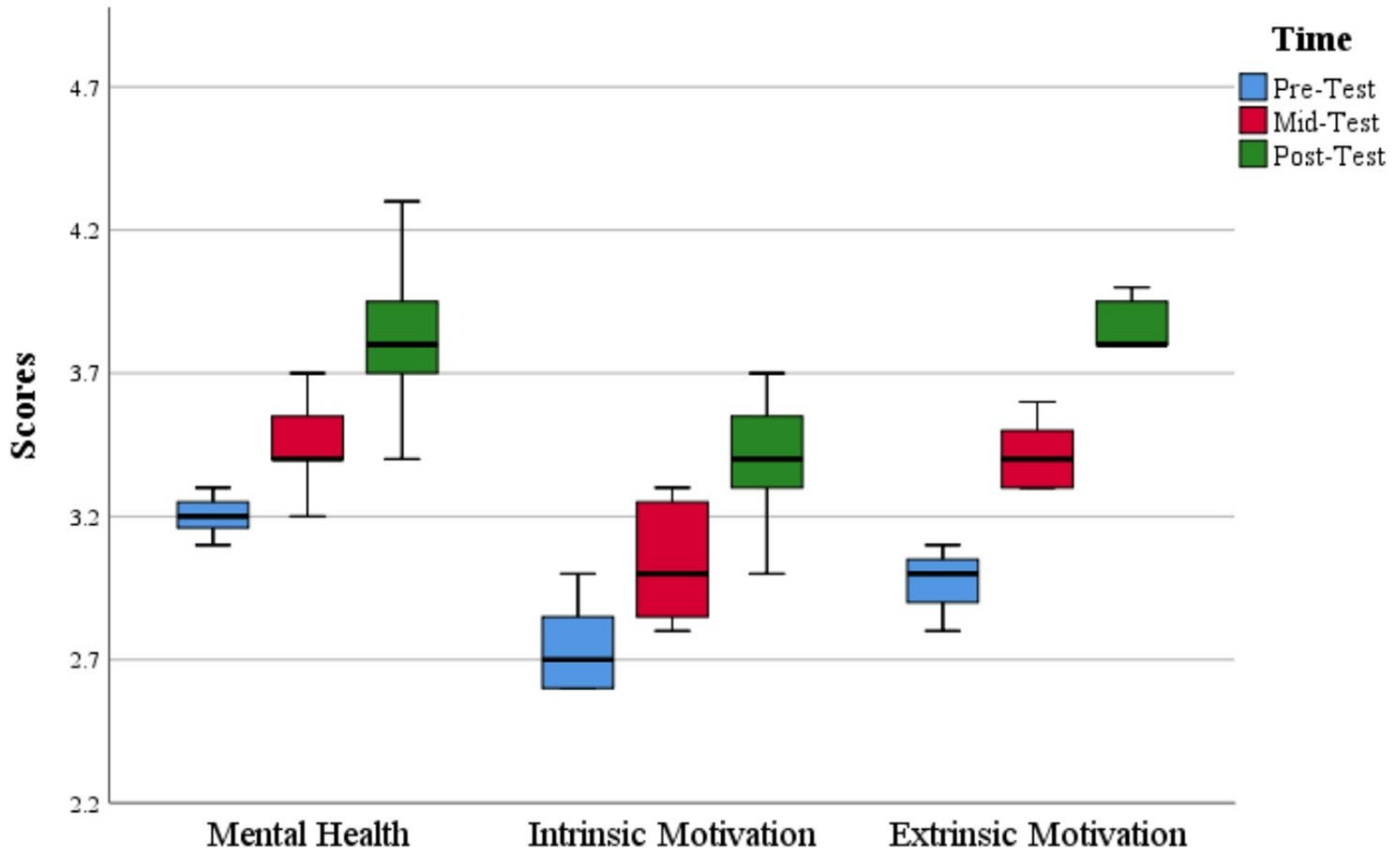
Box plot comparison

The differences among study dimensions at all time intervals were further illustrated by the box-and-whisker contrast (see Fig. 3). The plot shows a rising inclination in median values for all constructs, with MH demonstrating the greater upsurge across time, reflecting the efficiency of the intervention. Moreover, we observed a few outliers in the plot, which suggest that a small number of participants responded to the intervention in ways that deviated from the overall trends. These outliers may be attributed to certain individual differences such as prior psychological state, physical fitness, or inconsistent participation in the running routine [82]. These outliers, however, do not diminish the general patterns observed; they underscore the importance of considering individual differences in future interventions. For instance, future studies could use control variables or supplement with qualitative follow-ups to streamline the personal and contextual differences.

**Fig. 4:**
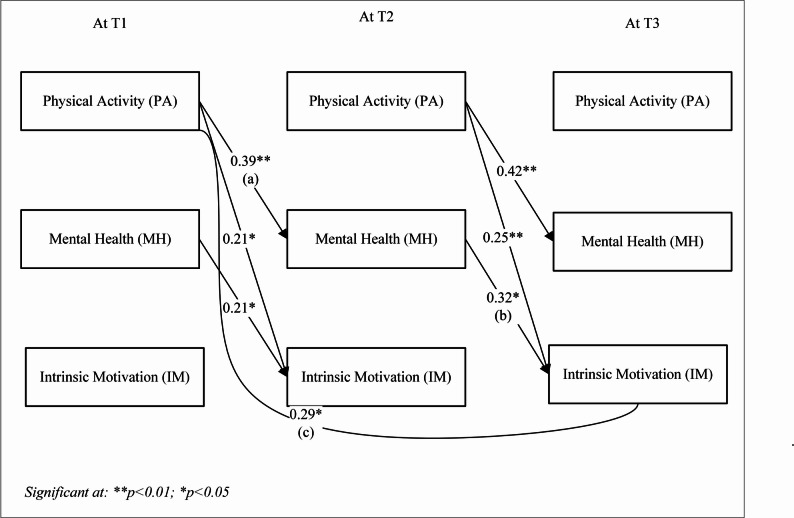
Model 1. longitudinal associations for intrinsic motivation

**Fig. 5:**
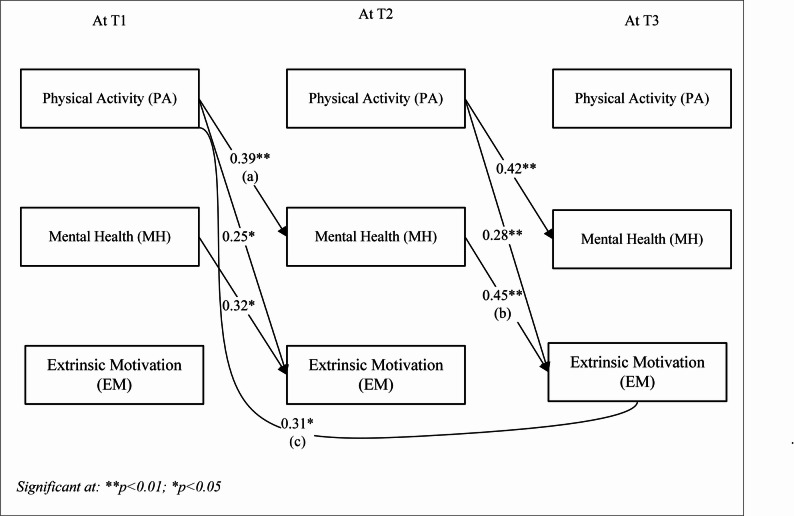
Model 2. longitudinal associations for extrinsic motivation

To examine the longitudinal mediating association of mental health between running exercise and students’ academic motivation, we employed Jose’s [83] prescribed procedure that involved SEM approach via a bootstrapping procedure with 5,000 resamples and 95% confidence intervals (see Figs. 4 and 5, and Table 6). The two resulting models demonstrated a good model fit for intrinsic motivation (χ²/df ratio 2.7, RMSR 0.67, RMSEA 0.71, CFI 0.93, GFI 0.95, AGFI 0.82, TLI 0.95 at 95% CI) and extrinsic motivation (χ²/df ratio 2.82, RMSR 0.65, RMSEA 0.68, CFI 0.94, GFI 0.96, AGFI 0.84, TLI 0.93 at 95% CI) as per Agostino’s [78] prescribed criteria. The results showed that mental health at T2 significantly mediated the relationship between PA at T1 and academic motivation for both intrinsic motivation (*ꞵ* = 0.12, *p* = 0.01) and extrinsic motivation (*ꞵ* = 0.17, *p* < 0.01) at T3.

**Table 6:** Mediation paths and coefficients

Path	ꞵ	SE	*p*-value
Intrinsic Motivation (IM)			
Physical Activity (PA_T1) → Mental Health (MH_T2) (a)	0.39	0.20	< 0.01
Mental Health (MH_T2) → Intrinsic Motivation (IM_T3) (b)	0.32	0.16	< 0.05
Physical Activity (PA_T1) → Intrinsic Motivation (IM_T3) (c)	0.29	0.15	< 0.05
Indirect effect (ab)	0.12	0.06	0.01
Total effect (ab + c)	0.41	0.21	< 0.01
Extrinsic Motivation (EM)			
Physical Activity (PA_T1) → Mental Health (MH_T2) (a)	0.39	0.20	< 0.01
Mental Health (MH_T2) → Extrinsic Motivation (EM_T3) (b)	0.45	0.23	< 0.01
Physical Activity (PA_T1) → Extrinsic Motivation (EM_T3) (c)	0.31	0.16	< 0.05
Indirect effect (ab)	0.17	0.09	< 0.01
Total effect (ab + c)	0.48	0.24	< 0.05

Concurrently, the results from model 1 (see Fig. 4) indicated that path a (the effect of physical activity at T1 on mental health at T2) and path b (the effect of mental health at T2 on intrinsic motivation at T3) were significant, with coefficients of *ꞵ* = 0.39 (*p* < 0.01) and *ꞵ* = 0.32 (*p* < 0.05), respectively. Additionally, the effect in path c, which examines the relationship from physical activity at T1 to intrinsic motivation at T3, was also significant in this model (*ꞵ* = 0.29, *p* < 0.05).

Model 2 (see Fig. 5) showed that path a (the effect of physical activity at T1 on mental health at T2) and path b (the effect of mental health at T2 on extrinsic motivation at T3) were both significant, with coefficients of *ꞵ* = 0.39 and 0.45, respectively (*p* < 0.01). Additionally, the effect in path c from physical activity at T1 to extrinsic motivation at T3 was also significant in this model (*ꞵ* = 0.31, *p* < 0.05).

## Discussion

Maintaining a high-quality education to meet global demands has been one of the central goals of higher education institutions. In the meantime, academic motivation is considered a key indicator of students’ academic progress [6]. Given this, their motivation to learn has received increased attention within the fields of psychology and education over the past decade [7, 11]. Moreover, mental health is one of the critical factors that determine students’ ability to engage with academic tasks and sustain their motivation to learn. As a result of their strong correlation with learning, abilities, tactics, and behaviors, academic motivation and mental health receive great attention [17, 19], and psychologists have proposed various strategies to help students enhance their academic motivation. Considering this, the current study explores the effects of running as a physical activity on the mental wellness and motivational dynamics of learners via the SDT framework.

We found evidence of a positive influence of physical activity on students’ MH, affirming the crucial role that PA plays in promoting psychological wellbeing in the educational setting. This insight corresponds with Bafageeh and Loux’s [84] reflection, which ascertains a positive effect of regular physical exercise on individuals’ MH status. Identifying types, they noticed that running, athletics, and lifting weights had a substantial correlation with mental health, while household chores exhibited a minimal correlation. Concurrently, it has also been observed that sedentary lifestyles and physical inactivity may exacerbate mental health conditions like anxiety and emotional instability [85]. Aligning with this observation, Ernstsen and Havnen [33] point out that exercise has an antidepressant effect through a number of physiological and psychological mechanisms that aid in managing stressful situations and preserve the mental well-being of an individual. This effect can be attributed to some other factors. Firstly, regular exercise enables students to establish a structured regimen that fosters self-discipline and the capacity for resilience [86]. Moreover, a routine of regular exercise also offers a variety of chances for social connections, which further contribute to mental wellness. Since social relationships are required for people’s good psychological functioning [87], it motivates them to engage in PA to build interpersonal contacts. Hence, embedding various forms of structured physical activities that foster students’ sense of self-discipline and social interactions in educational programs is recommended to strengthen their mental wellness. Institutions should also construct a safe running track or a space for physical activities in order to give students a safe environment. These activities are preferred to be undertaken in groups in order to develop a sense of community among students and a rich, socially supportive environment. Consultants may also suggest individual-based workouts to address their mental and physical health requirements.

The findings further indicate that the running practice significantly increases students’ intrinsic motivation to learn. This insight corresponds with Vazou et al.’s [88] reflection, which ascertains a positive effect of regular exercise on pupils’ motivation to learn intrinsically. Aligning this observation, Young-Jones et al. [31] highlight how exercise may substantially boost college students’ motivation and self-confidence. This observation lends support from Deci and Ryan’s [39] SDT proposition, which demonstrates how autonomy, competence, and relatedness may improve an individual’s IM. Students are expected to be experienced and self-directed upon engaging in an organized exercise routine, which could help them elevate their IM. Broadening this notion, Rahayu et al. [89] identified an important role of perceived competence and autonomy in shaping students’ IM. Hence, utilizing various forms of physical activities such as individual, group based, and traditional sports that foster students’ core motivational needs would be highly beneficial. To achieve this, institutions should provide students with opportunities to lead and manage the sports programs they are passionate about. According to SDT [39, 56], this approach would enhance their sense of autonomy, increasing their interest and enabling them to perform in a more meaningful way. Moreover, institutions should offer beginner-friendly sports workshops to gradually build students’ competence, with instructors praising them for small wins. When students feel that they are improving, they are more likely to stay motivated. Moreover, academic learning platforms should be integrated with physical activities. This approach is expected to help students re-energize their focus, enhance their learning engagement, and strengthen their motivation to participate in both academics and physical activities.

We further observed a strong influence of running practice on students’ extrinsic motivation, which remained greater than IM at all time points. Correspondingly, this finding indicates the importance of external factors serving to facilitate motivation amongst students. This trend indicates that students seek tangible outcomes for their educational pursuits, such as good grades or preparation for good professional opportunities. This finding aligns with Ryan and Deci’s [5] conceptualization, where they imply that prioritizing external benefits may cause individuals to outweigh their IM. Aligning with this notion, Kusumawati et al. [90] identify that when students give preference to gaining good grades, they don’t find their learning process enjoyable. Schunk [7] also acknowledges that students should have a better experience in learning without the stress of giving good grades or rewards and identify the importance of IM in attaining long-term goals. In the meantime, Chang et al. [91] imply external stimuli as a matter of urgency in engaging students actively in their studies. In another study, Suman [92] points out the importance of extrinsic motivators in attaining academic objectives. Diversifying its importance, EM is positively linked with the self-efficacy of individuals, which aligns with the principles of social cognitive theory demonstrated by Schunk and DiBenedetto [6]. Given that, self-regulated learning skills should be promoted among students to provide them with opportunities for competence development and the appropriate support to increase their self-efficacy. Moreover, Academic authorities can leverage external incentives by providing awards or accolades to students who consistently participate in PA programs. This strategy aims to boost their motivation and increase their active involvement in such programs.

The mediating role of MH further contributes to an in-depth insight into the ways in which PA influences students’ AM over time. The findings indicate that MH served as a significant mediator between PA and academic (IM and EM) motivation. This finding resonates with Rebar and Taylor’s [85] observation on the multifaceted function of physical activity on individuals by improving serotonin and dopamine levels, and reducing cortisol levels. This process improves an individual’s psychological and physical well-being by lowering stress, controlling emotions, and boosting self-efficacy. Psychological wellness is also regarded as a necessary precondition for SDT since it enables students to undertake challenging assignments on their own and scaffolds their learning. Its conceptualization also suggests that the psychological well-being of an individual is considered a prerequisite of sustained motivation [39]. Supported by this belvedere, students with better MH are expected to engage in tasks actively and show satisfactory academic performance. Comparing across types, we found that physical activity had a stronger indirect impact on extrinsic motivation than intrinsic motivation. It indicates that students are more driven by external factors than by internal enjoyment or curiosity. On one hand, this finding demonstrates the value of EM in attaining academic objectives [92]. At the same time, Morris et al.’s [93] observation points out a negative effect of extrinsic motivator factors on students’ MH. It is further supported by the fact that relying too much on external incentives can cause stress and anxiety if they don’t receive them frequently, which affects their psychological health. Fostering intrinsic motivation, on the other hand, is usually considered to be preferable for MH and sustainable learning outcomes. In light of this, teachers should look for a balance between the external benefits and opportunities to help them find internal motivation. To do so, learning environments should adopt a dual-strategy approach that balances both motivational types. For instance, extrinsic rewards can initially drive student engagement towards physical activities, while SDT-integrated PA approaches would support students’ IM towards learning by fulfilling students’ core psychological needs [39]. To support students’ autonomy, their preferences should be prioritized to foster a sense of ownership. To help students feel competent, they should be given challenging running goals and be allowed to set their own goals, along with receiving constructive feedback. To address relatedness needs, tasks should be distributed to students in groups to foster social interactions. Additionally, teachers should appreciate them appropriately and support emotionally to help meet this need. They can also integrate project-based techniques into their pedagogical practices to allow students to ask questions to clear their confusion. Furthermore, students should be given feedback that focuses on their learning processes rather than the product itself. This can help them create a stronger connection to the material, which can increase their intrinsic motivation to learn.

Although the findings of the present study advance our knowledge about how PA facilitates learners’ mental and motivational behaviors, a few of the limitations are also recognized. Firstly, the research used a convenient sampling which was restricted to one university, due to which results cannot be generalized adequately to other contexts. In future studies, participants from various institutions or cultural backgrounds, employing other sampling procedures, should be recruited to better investigate the overview of the PA. Moreover, students’ physical activity involvement, motivational behaviors, and mental wellness may vary across different cultural or educational contexts. This emphasizes the need for future research to explore these findings in diverse cultural settings to enhance their broader applicability. Another weakness is that this study focused solely on running and lacked a control group, which limits interpretation and does not account for the effects of other physical activities. Future studies should therefore investigate the effects of different forms of physical activity, such as yoga or team sports, using control groups to examine the differences. Additionally, it is recommended to design studies that include both intervention and control groups to effectively isolate the effects.

## Conclusions

This study provides a comprehensive analysis regarding students’ motivational dynamics, focusing the role of physical exercise, mental health, and the SDT’s principles in shaping these pathways. While both intrinsic and extrinsic motivation increased over time, extrinsic motivation consistently remained higher across all time intervals. The analysis further illustrates a significant role of physical activity through mental health on academic motivation, revealing a greater mediation effect on EM compared to IM. This dominance suggests that students are more driven by external rewards and pressures than by the inherent satisfaction derived from learning. Consequently, the study emphasizes the necessity of fostering student motivation through the integration of psychological support alongside participation in physical activity programs to achieve holistic wellness. Furthermore, the consistently higher levels of extrinsic motivation imply that students’ intrinsic needs, according to SDT principles, may not be adequately addressed in the current educational environment. The results underscore the importance of educational institutions implementing strategies that not only bolster extrinsic motivators but also promote intrinsic motivation through practices that prioritize autonomy, competence, and personal growth. Tailored interventions that address the specific motivational needs of students in their academic journeys are crucial for enhancing overall academic outcomes.

## Data Availability

The datasets generated and analyzed during the current study are available from the author on reasonable request.
